# Detentions in BSMHFT (Birmingham and Solihull Mental Health Foundation NHS Trust) - Covering the Birmingham and Solihull Geographical Area Under the Mental Health Act Between 2018 to 2021

**DOI:** 10.1192/bjo.2022.393

**Published:** 2022-06-20

**Authors:** Dinesh Maganty, Rajendra Harsh, Tom Cleverley

**Affiliations:** Birmingham and Solihull Mental Health Foundation NHS Trust, Birmingham, United Kingdom

## Abstract

**Aims:**

To continue to monitor trends in detentions under the Mental Health Act based on race, age, gender, and sexuality during the COVID-19 Pandemic to consider if there were any specific areas that would need to be addressed.

**Methods:**

We investigated available mental health detention documents stored in mental health legislative office, Birmingham and Solihull mental health foundation NHS Trust.

**Results:**

We found that detentions under Section 3 of the Mental Health Act have increased very gradually over the last three years (2018 to2021). However, there has been gradual reduction in detentions under Section 3 within the white population beginning in 2019 and continuing with a marked acceleration in reduction during the two peaks of the pandemic. This is marked in the 66yrs plus age group. As the pandemic has eased this reduction has stopped and reversed with increased section 3 admissions in last few months in this population. The detentions in the black and Asian population have followed a reverse pattern, with marked increase during the pandemic peaks in 2020/2021 and a marked fall as the pandemic has eased.

**Conclusion:**

1. Mental health act detention data during the Pandemic shows that the pandemic has disproportionality impacted black and Asian population of all ages and Elderly white population.
2. During the pandemic there has been a marked increase in detentions under Section 3 of the Mental Health Act (for treatment) in the Black and Asian population with a marked reduction in the white population. This difference is stark in the working age population.
3. This highlights:
  - The need for a well-functioning community based health and social care offer to reduce detentions in the black and Asian population.
  - Return of admissions under the mental health act of white elderly post vaccination (which are vast majority white) shows a reversal of the trend of this group not accessing inpatient treatment fully during the pandemic.
4. Community Treatment Order (CTO) detentions in the Black and Asian population continue to increase through the pandemic disproportionately
5. There is no material change during the pandemic, in short term detentions (section 2, 5(2)) or other inpatient detentions under the Mental health act
6. There are no significant trend changes noted based on gender or sexuality or age during the pandemic in BSMHFT (Birmingham and Solihull mental health foundation NHS Trust).

